# A Six‐Step Approach for Optimizing Ultrasound‐Guided Femoral Artery Access

**DOI:** 10.1002/ccd.31646

**Published:** 2025-06-02

**Authors:** Fernando Luiz de Melo Bernardi, Guilherme Luiz de Melo Bernardi, Júlio Roberto Barbiero, Adalberto Gloeckner de Meira, Luiz Felipe Monsanto Fernandes Alves, Dinaldo Cavalcanti de Oliveira, Estevão Carvalho de Campos Martins

**Affiliations:** ^1^ Hospital Unimed Chapecó Chapecó Santa Catarina Brazil; ^2^ Hospital Regional do Oeste Chapecó Santa Catarina Brazil; ^3^ Hospital da Força Aérea do Galeão Rio de Janeiro Brazil; ^4^ Universidade Federal de Pernambuco Recife Brazil

**Keywords:** arterial femoral access, common femoral artery, ultrasound‐guided access

## Abstract

Femoral arterial access is fundamental to interventional cardiology, especially for complex procedures that require large‐bore devices. However, vascular complications arising from suboptimal puncture techniques highlight the need for meticulous practice. This article presents a systematic, ultrasound‐guided approach for achieving an optimal common femoral artery (CFA) puncture, defined by five key criteria: access within the CFA, anterior wall‐only penetration, centralized lumen entry, puncture above the femoral head, and entry into a healthy arterial segment. We detail a six‐step protocol incorporating ultrasound guidance throughout: (1) identification of the CFA and femoral vein; (2) delineation of CFA boundaries; (3) localization of the femoral head; (4) determination of the optimal puncture site; (5) real‐time needle tracking and precise CFA puncture; and (6) confirmation of correct guidewire insertion. This protocol enhances both the safety and reproducibility of the procedure. Although there is a significant learning curve, proficiency with this method aims to enable rapid, consistent, and low‐complication vascular access. The article offers practical recommendations for implementation in the catheterization laboratory—including equipment selection and team training—and reviews current literature supporting the efficacy of ultrasound in minimizing access‐related complications.

## 1 Introduction

Femoral arterial access continues to occupy a central role in interventional cardiology, despite the growing adoption of radial access. Its utility is most pronounced in procedures requiring large‐bore sheaths, such as transcatheter aortic valve replacement (TAVR), endovascular aneurysm repair, and mechanical circulatory support deployment. Data from registries indicate that femoral access accounts for approximately 40%−50% [1, 2] of coronary interventions and over 90% of structural heart procedures in contemporary practice [3, 4]. However, this approach is not without risks. Vascular complications—ranging from minor hematomas to life‐threatening bleedings—occur in 1%−10% of cases, with rates escalating in patients with obesity, anticoagulation, or peripheral artery disease [5].

The cornerstone of minimizing these complications lies in achieving a “perfect” common femoral artery (CFA) puncture. Traditional landmark‐based techniques, reliant on palpation of the femoral pulse and fluoroscopic guidance, are inherently imprecise, with studies reporting misplacement rates of 20%−30% [6]. Such errors often result in “high sticks” into the external iliac artery (EIA), risking retroperitoneal bleeding, or “low sticks” at or below the CFA bifurcation, predisposing to pseudoaneurysms or arteriovenous fistulae. Ultrasound guidance has emerged as a transformative tool, offering real‐time visualization of vascular anatomy and needle trajectory. Its adoption has been associated with reduced complication rates, higher first‐pass success, and improved patient outcomes [7, 8, 9].

This article proposes a systematic, six‐step protocol for ultrasound‐guided CFA puncture, designed to meet rigorous criteria for a “perfect” puncture utilizing standard ultrasound equipment. The protocol enables operators to consistently perform high‐quality punctures without requiring advanced mastery of ultrasound techniques. By integrating anatomical precision with practical considerations for the catheterization laboratory, this approach aims to standardize femoral access, mitigate risks, and enhance procedural efficiency.

## 2 Defining the Perfect CFA Puncture

**Figure 1:**
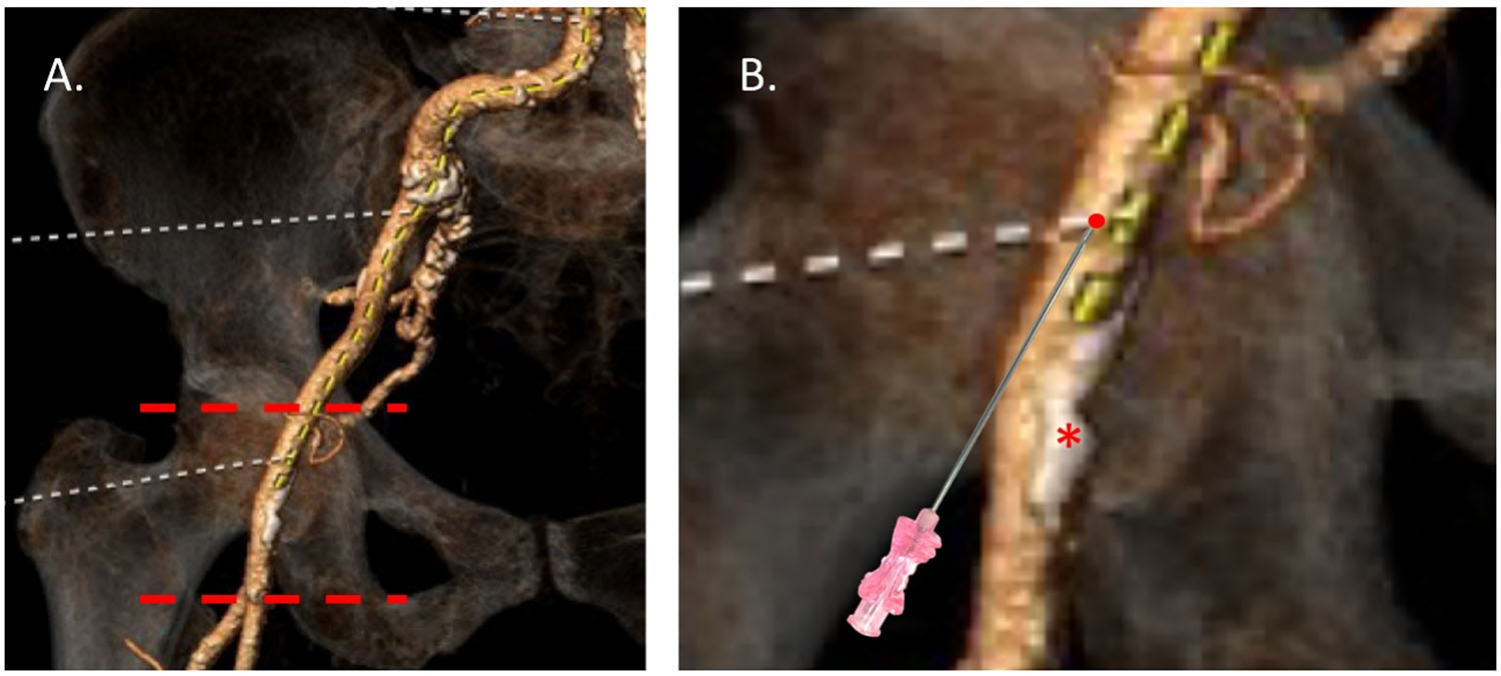
The perfect CFA puncture. Location of a perfect CFA puncture in a patient planned for TAVR. Image (A) shows the boundaries of the CFA. The CFA begins immediately after the inferior epigastric artery, where the external iliac artery exits the retroperitoneal space. It passes beneath the inguinal ligament and extends up to the bifurcation point, where it divides into the superficial femoral artery and the profunda femoris artery. (B) Illustration depicting the optimal site CFA puncture (red dot). Ideally, the puncture should occur approximately 2 cm away from the artery's boundaries. It should be centered, avoiding any calcified segments (*) and situated directly over the femoral head. Furthermore, the puncture should be a single entry through the anterior wall of the artery. [Color figure can be viewed at wileyonlinelibrary.com]

A “perfect” CFA puncture (Figure 1) is a procedural ideal that minimizes vascular complications and optimizes subsequent steps, such as sheath insertion and closure. It is characterized by five essential criteria:

- Puncture within the CFA: The needle must target the CFA, avoiding a proximal puncture into the EIA (above the inguinal ligament, risking retroperitoneal hemorrhage) or a distal puncture at or below the CFA bifurcation (into the superficial or profunda femoris arteries, increasing pseudoaneurysm risk).
- Anterior wall penetration: The puncture should involve only the anterior wall, preventing “back‐wall” transgression that may cause uncontrolled bleeding or hematoma formation.
- Centralized lumen access: The needle should enter the center of the arterial lumen (at 12 o'clock), ensuring optimal sheath positioning and reducing eccentric vessel trauma.
- Position above the femoral head: The puncture site must overlie the femoral head, providing a solid bony backstop for manual compression if hemostasis is required, especially in anticoagulated patients.
- Healthy arterial segment: The target zone should be free of atherosclerotic plaque, particularly calcified lesions, which can hinder suture‐based vascular closure devices (e.g., Perclose ProGlide) and increase risks of embolization or occlusion.

These criteria address the anatomical and technical challenges of femoral access, aligning with the goal of reducing complications that compromise patient safety and prolong recovery. Ultrasound guidance uniquely facilitates adherence to these standards by enabling precise visualization of the CFA and its surrounding structures.

## 3 Systematic Approach to Ultrasound‐Guided CFA Puncture

The proposed six‐step protocol ensures a safe, reproducible CFA puncture, achieving the “perfect” CFA puncture outlined above. Each step leverages ultrasonography to enhance precision, with technical nuances tailored to patient‐specific challenges.

### 3.1 Step 1: Differentiation of the CFA and the Femoral Vein

**Figure 2:**
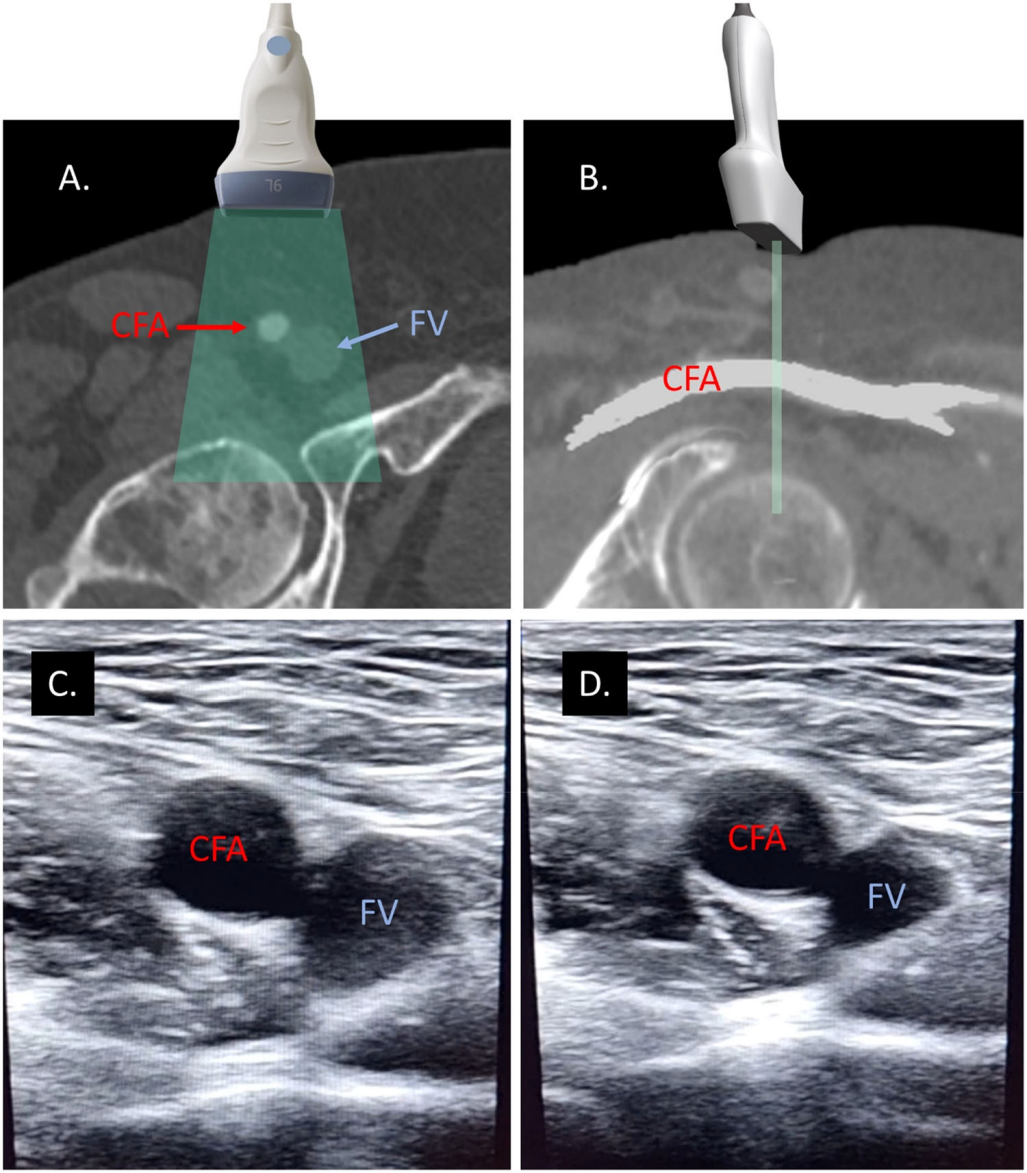
Differentiating the common femoral artery and femoral vein (Step 1). In this initial step, the focus is on distinguishing between the common femoral artery (CFA) and the femoral vein (FV). (A) and (B) Illustrate the correct transducer orientation necessary for obtaining a transverse ultrasonographic view of these vessels. Images (C) and (D) depict the ultrasonographic view of the CFA and FV before and after applying pressure with the transducer, respectively. The application of pressure demonstrates the compressibility of the FV, aiding in its differentiation from the CFA. [Color figure can be viewed at wileyonlinelibrary.com]

To initiate the procedure, a high‐frequency linear transducer (7−12 MHz) is placed inside a sterile bag. The skin is then moistened with sterile saline. The transducer is positioned transversely over the inguinal region to achieve optimal imaging of the femoral vessels. It's crucial to ensure there is no air trapped between the sterile bag and the transducer (we always cover the transducer tip with ultrasound gel before encasing it in the bag). The CFA is identified as a pulsatile, hypoechoic structure lateral to the compressible femoral vein. This transversal view can also serve for screening for anatomical variants (e.g., high bifurcation) and confirms the CFA's position relative to the vein, typically medial. Gentle pressure distinguishes the noncompressible artery from the vein, ensuring accurate initial orientation (Figure 2 and Video S1).

### 3.2 Step 2: Identification of the CFA Boundaries

**Figure 3:**
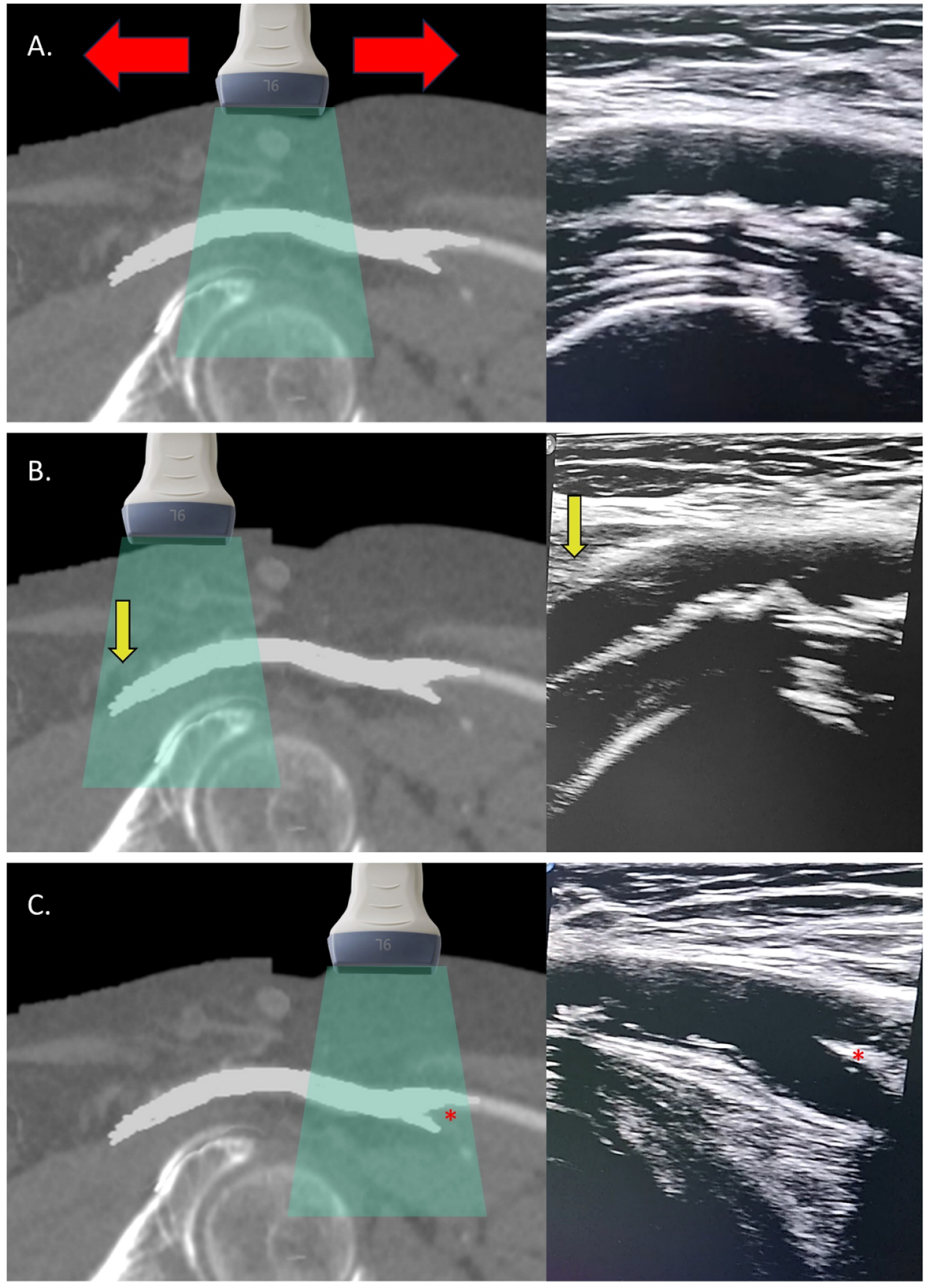
Identification of the CFA boundaries (Step 2). (A) Positioning: The operator initially aligns the probe longitudinally with the CFA. The operator then scouts the artery by moving the probe cranially (B) and caudally (C) to capture the full extent of the CFA, effectively identifying its anatomical boundaries. Yellow arrows highlight the critical point where the CFA dips beneath the inguinal ligament, transitioning into the EIA within the retroperitoneal space. Red asterisks denote the bifurcation of the CFA into the superficial femoral artery and the profunda femoris artery, clearly marking the division points. [Color figure can be viewed at wileyonlinelibrary.com]

The transducer is rotated close to 90° to obtain a longitudinal view of the CFA. Usually, the CFA courses in 20−25° angle from the sagittal plane, so the probe must be slightly oriented medially. The operator should keep the probe at a 90° angle from the skin, avoiding tilting it, and running the probe cranially and caudally to visualize the CFA's full course. Proximally, the EIA emerges from the retroperitoneum, becoming superficial as it transitions into the CFA beneath the inguinal ligament (where the inferior epigastric artery emerges). Distally, the CFA bifurcates into the superficial and profunda femoris arteries. Proper alignment of the ultrasound beam parallel to the arterial axis is critical for clear imaging. This step defines the CFA's boundaries, ensuring the puncture site avoids the EIA or the bifurcation (Figure 3 and Video S1). This is also the moment to visually assess the diameters of the CFA and the presence of atherosclerotic plaques.

### 3.3 Step 3: Femoral Head Visualization

**Figure 4:**
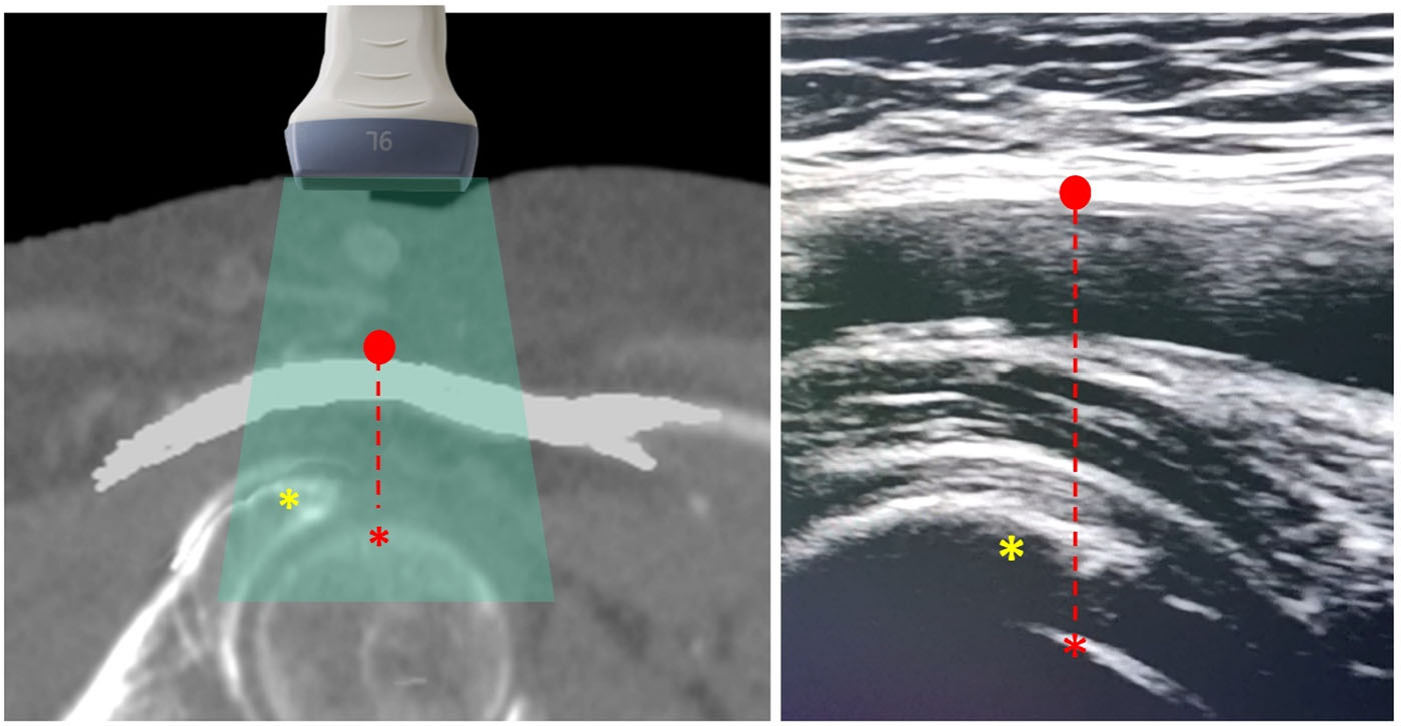
Identification of the femoral head (Step 3) and defining the ideal CFA puncture site (Step 4). Ultrasound imaging has been utilized to identify key anatomical structures such as the femoral head, which is indicated by a red asterisk, and the acetabulum, marked with a yellow asterisk. For optimal procedure outcomes, the ideal puncture site for the anterior wall of the CFA should be located above the femoral head, at a distance of at least 2 cm from the CFA boundaries, and ensuring the area is free from disease (red dot). [Color figure can be viewed at wileyonlinelibrary.com]

The femoral head and the acetabulum are hyperechoic, curved structures, identified beneath the CFA. In slim patients, they lie 2−3 cm deep; in obese patients, depth settings must be adjusted (4−6 cm) to penetrate subcutaneous fat. Also, the operator can compress the subcutaneous tissue with the probe to shorten the distance between the femoral head/acetabulum and the skin. This bony landmark confirms the puncture site's compressibility, a key safety feature. The operator may slide the probe cranially or caudally to align the CFA's mid‐segment with the femoral head's apex (Figure 4 and Video S1).

### 3.4 Step 4: Target Selection

An ideal puncture site is selected on the CFA's anterior wall, typically 2 cm distal to the inguinal ligament and 2 cm proximal to the bifurcation. The operator scans for diseased segments of the anterior wall, identified as hyperechoic irregularities (plaque) or shadowing (calcification). Avoiding these ensures compatibility with closure devices and reduces procedural risks. The target is marked mentally on the ultrasound screen. Once a proper ultrasonographic window is achieved, the operator must lock his/her left hand which holds the probe to keep a still image of the CFA (Figure 4).

### 3.5 Step 5: Needle Tracking

**Figure 5:**
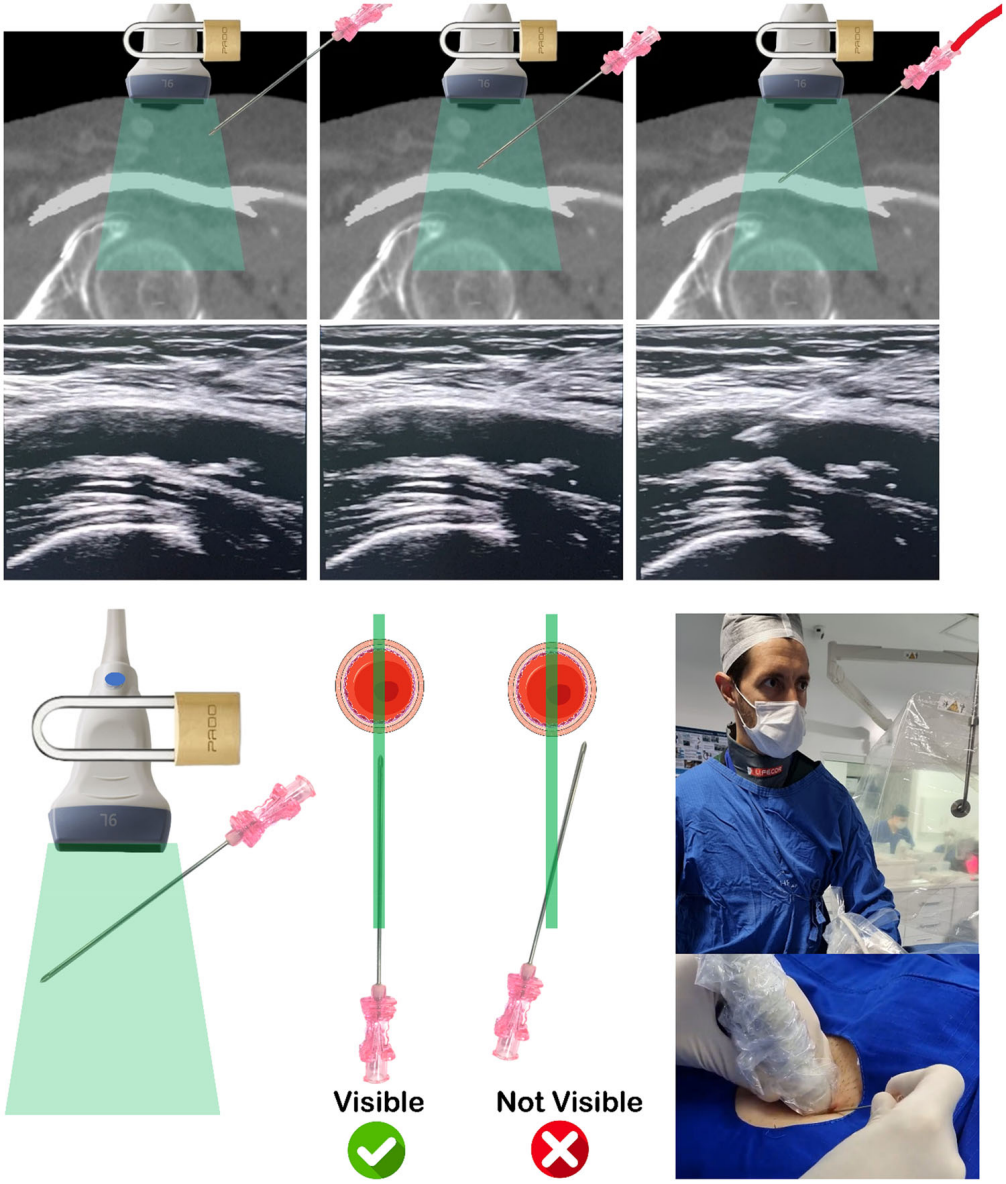
Real‐time ultrasound needle tracking (Step 5). Once achieved an optimal ultrasonographic view of the common femoral artery (CFA), the operator should secure the left hand in place, as demonstrated by the lock illustration. From there on, only the right hand, holding the needle, should move. The operator must keep their eyes on the ultrasound screen at all times. For successful visualization, the needle must be precisely aligned with the ultrasound beam to appear in the image. It should be inserted 1−2 cm into the subcutaneous tissue to be visible. Only advance the needle once its tip is clearly seen. For patients with minimal subcutaneous tissue, an alternative technique may be preferable, as shown in Figure 6B. If the needle is not visible, it indicates misalignment with the ultrasound beam. In such cases, the operator should not advance the needle. Instead, they should adjust the needle sideways or withdraw and reinsert it to ensure alignment and visibility. If the operator struggles to maintain alignment of the needle tip with the ultrasound beam as it nears the CFA—resulting in a loss of needle visualization on the screen—they can rotate the transducer to a transverse view. This adjustment helps ensure a central puncture of the vessel, as shown in Figure 6A. [Color figure can be viewed at wileyonlinelibrary.com]

With the transducer held steady at 90° to the skin using the left hand, the right hand introduces an 18‐gauge needle parallel and adjacent to the probe. Live longitudinal imaging tracks the needle through the subcutaneous tissue. The needle is visible only when aligned with the ultrasound beam, which is as thin as a credit card (~1 mm). Once a perfect acoustic window is established, the left hand remains immobile, and all subsequent movements are performed with the right hand. The needle enters the skin 1−2 cm from the probe, advancing only when visualized on the ultrasound screen. Eyes remain fixed on the monitor, not the puncture site (Video S2 and Figure 5).

**Figure 6:**
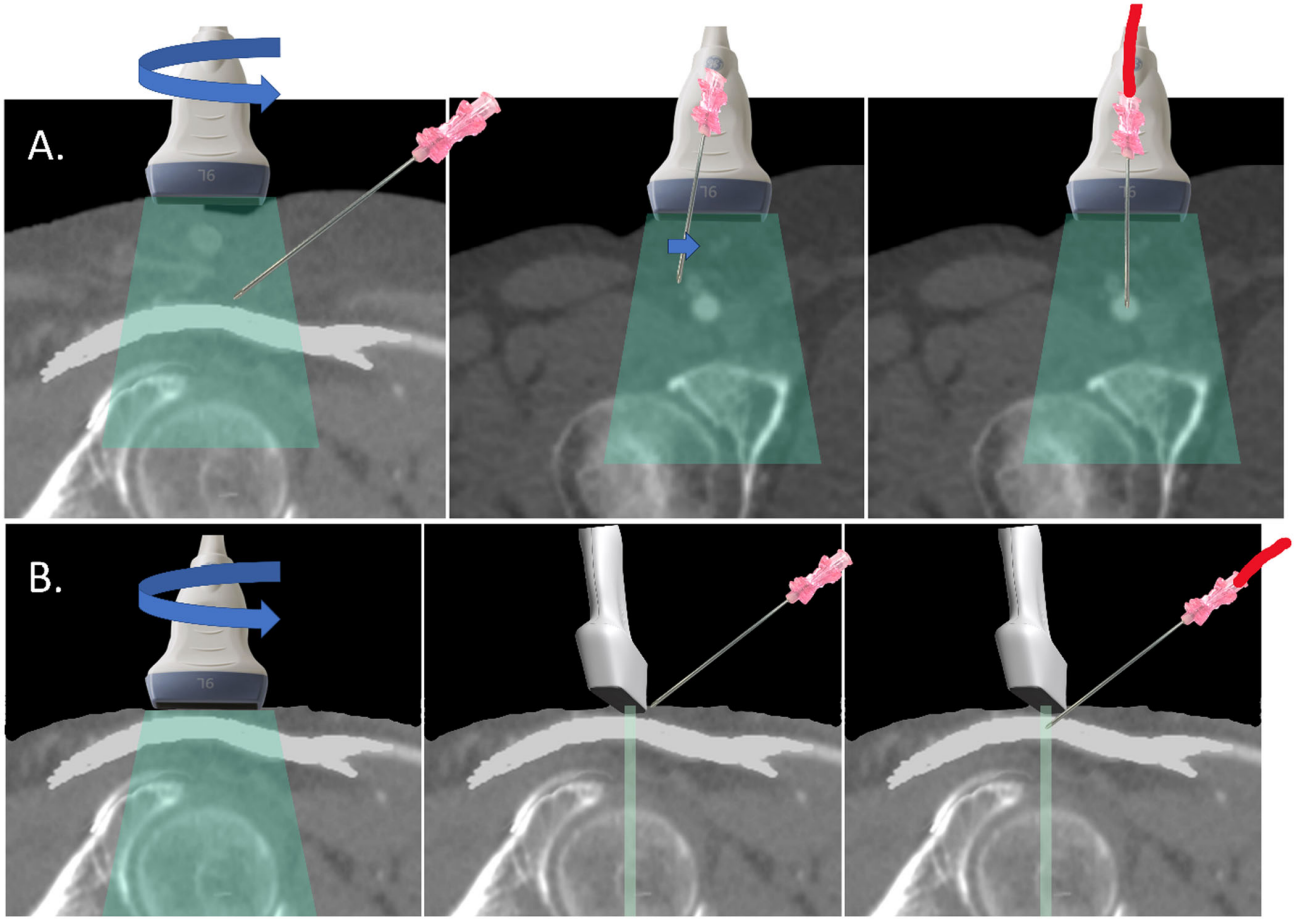
Alternative techniques for ultrasound‐guided CFA puncture. (A) When the operator struggles to maintain alignment of the needle tip with the ultrasound beam as it nears the CFA—resulting in a loss of needle visualization on the screen—they can rotate the transducer to a transverse view. This adjustment helps ensure a central puncture of the vessel. (B) In patients with very thin subcutaneous tissue (less than 1–2 cm), where there is insufficient space to visualize and maneuver the needle in the longitudinal ultrasonographic view, it is preferable to place the ultrasound probe directly over the chosen puncture site in a transverse orientation to the common CFA. The needle should then be advanced perpendicularly at a 45° angle, targeting the center of the artery. [Color figure can be viewed at wileyonlinelibrary.com]

If the needle disappears, gentle back‐and‐forth adjustments (< 1 cm) correct its trajectory until it reappears. As the needle nears the artery, its tip must be clearly seen; if obscured, the transducer can be rotated to a transversal view to centralize the tip within the lumen (Figure 6A). In thin patients with minimal subcutaneous tissue, tracking is challenging due to limited correction space. Here, after identifying the target in longitudinal view, the operator rotates to a transversal view directly over the site, advancing the needle under this plane. The needle punctures the anterior wall, confirmed by pulsatile back bleeding (Figure 6B).

### 3.6 Step 6: Confirmation of Correct Guidewire Insertion

**Figure 7:**
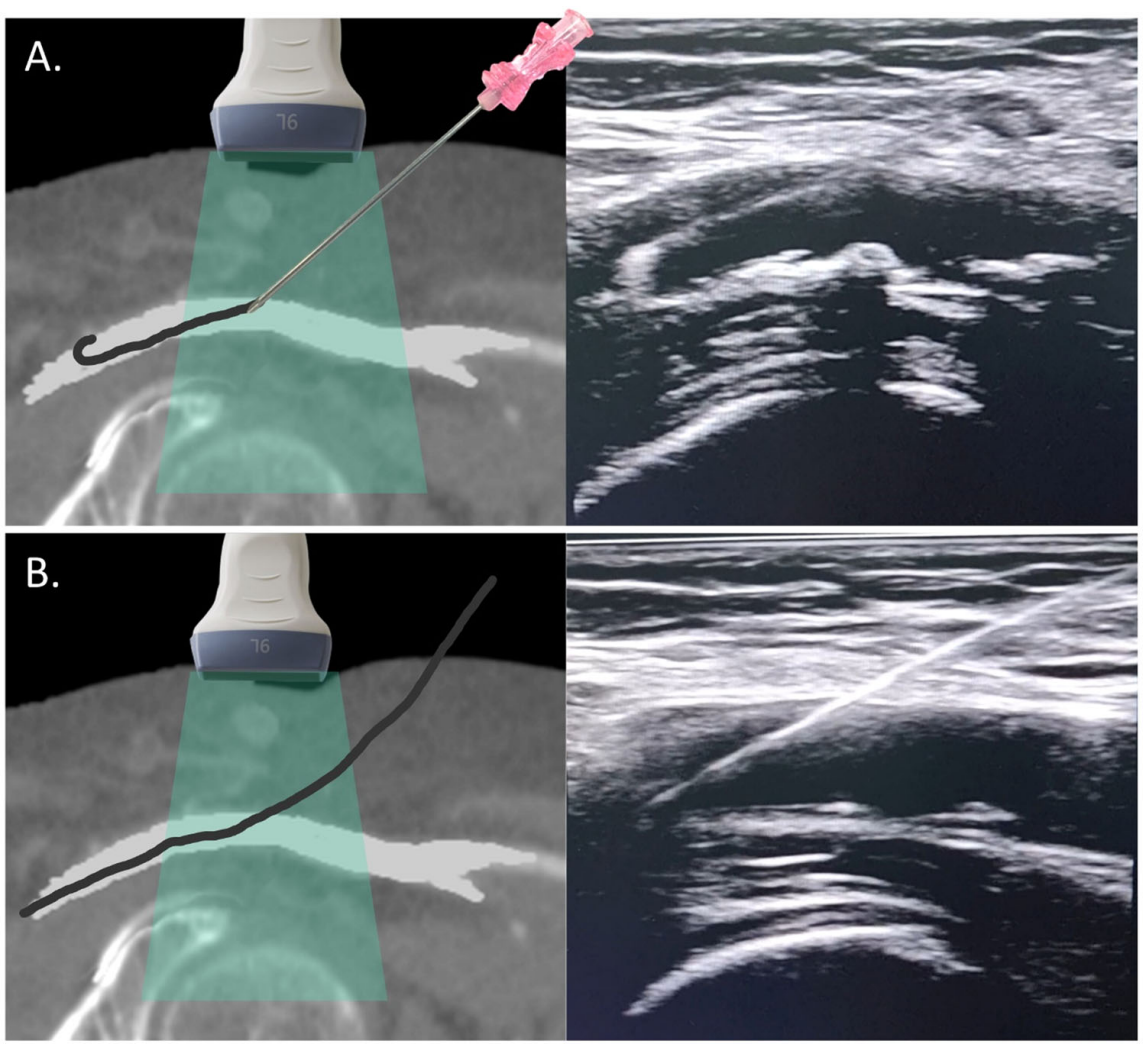
Confirmation of guidewire position in the true lumen and entry point (Step 6). (A) After successfully puncturing the CFA anterior wall, the operator can visualize in real‐time the guidewire advancement of the guidewire into the true lumen of the vessel under ultrasound guidance. (B) Following needle removal, the operator uses ultrasound to reassess the CFA at the puncture site, ensuring proper location and vessel integrity before sheath insertion. [Color figure can be viewed at wileyonlinelibrary.com]

After CFA puncture, without moving the left hand, maintaining the ultrasound on longitudinal view, a 0.035” guidewire is advanced through the needle into the CFA under direct visualization. The wire's echogenic tip should traverse the true lumen, avoiding side branches or dissection planes (Video S2). Video S3 showcases the technique of a single operator advancing the guidewire into the CFA under real‐time ultrasound guidance. After needle removal, a longitudinal scan confirms the wire's position and entry point (Figure 7). If too high (near the EIA) or too low (near the bifurcation), the wire is withdrawn before dilation or sheath insertion, allowing repositioning.

## 4 Multimodality Imaging as a Tool for Calibrating Operator Hand

During the authors' initial learning curve with ultrasound‐guided puncture, the arterial entry point was frequently positioned slightly higher in the CFA than originally intended. We believe that operators adopting this technique should engage in a process of “self‐calibration” to avoid high arterial punctures and reduce the risk of retroperitoneal bleeding. In our current practice, we train fellows to calibrate their hand positioning and technique using multimodal image analysis—including angio‐CT, fluoroscopy, and ultrasound—to enhance both accuracy and safety.

**Figure 8:**
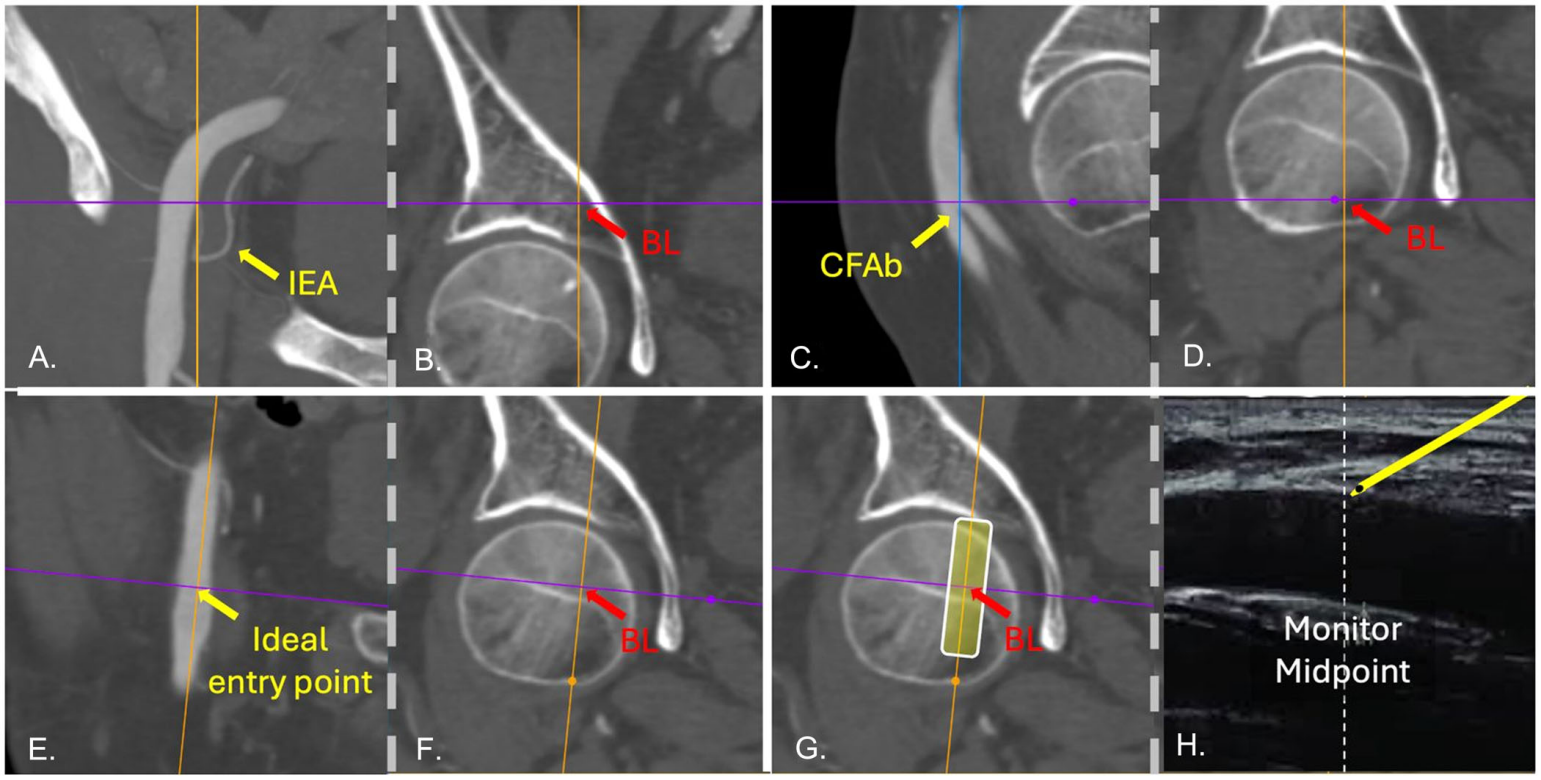
Multimodality imaging to aid ultrasound‐guided CFA puncture. (A) Positioning the cursor at the inferior epigastric artery (IEA) origin. (B) After scrolling the cursor at the coronal plane, the level of the IEA is marked in the femoral bone/acetabulum. (C) Positioning the cursor at the level of the common femoral artery bifurcation (CFAb) in the sagittal plane. (D) After scrolling the cursor at the coronal plane, the level of the CFAb is marked in the femoral bone. (E) The cursor is positioned at the level of the ideal entry point in the CFA. (F) After scrolling the cursor at the coronal plane. [Color figure can be viewed at wileyonlinelibrary.com]

For example, in patients undergoing TAVI, we begin vascular access planning using angio‐CT. First, we identify the origin of the inferior epigastric artery in the coronal plane (Figure 8A) and track this reference down to the level of the femoral bone, noting its anatomical correspondence (Figure 8B). This marks the upper boundary for femoral puncture. Next, we locate the bifurcation of the CFA in the sagittal view (Figure 8C) and similarly scroll this mark in the coronal plane down to the femoral bone (Figure 8D). The ideal arterial entry point is then identified and marked (Figure 8E), with its position again confirmed relative to the femoral bone (Figure 8F). With these steps, we establish both the superior and inferior limits for arterial puncture, as well as the optimal entry point, all referenced to bony landmarks.

During the procedure, we typically position the ultrasound probe over the precise puncture site determined on the femoral head (Figure 8G). On the ultrasound screen, it's important to ensure that the needle approaches from the right, targeting the center of the artery, rather than advancing toward the left side. This approach helps ensure that the trainee operator does not inadvertently puncture at a higher‐than‐desired location.

## 5 Additional Practical Tips

Successful integration of ultrasound‐guided access requires:

Equipment availability: Ensure an ultrasound system equipped with a linear probe (e.g., portable units with vascular presets) is accessible full‐time in the cath lab.

Team familiarization: Train staff to operate and troubleshoot ultrasound devices, fostering a collaborative workflow.

Sterile supplies: Stock sterile transducer covers and gel to maintain procedural sterility.

Complication detection skills: Enhance team proficiency in identifying hematomas, pseudoaneurysms, or dissections with ultrasound.

Practice opportunities: Leverage every femoral case to refine technique, building confidence and competence.

These measures bridge the gap between theoretical benefits and practical implementation, embedding ultrasound guidance into routine practice.

## 6 Literature Review

Current evidence strongly endorses ultrasound‐guided femoral access. The FAUST trial (2012) demonstrated higher first‐pass success (83% vs. 46%, *p* < 0.0001), improved CFA cannulation in patients with high bifurcations (82.6% vs. 69.8%, *p* < 0.01), and fewer venous punctures (1.4% vs. 9.8%, *p* < 0.0001) [7]. A 2020 propensity‐matched comparison showed a significant reduction in vascular complications and severe bleeding with ultrasound femoral access guidance in patients undergoing TAVR [10]. A meta‐analysis (2020) of seven RCTs enrolling 3180 patients showed a higher success rate of first attempt (82.0% vs. 58.7%; *p* < 0.00001), reduced time to access, number of attempts, vascular complications (1.3% vs. 3.0%; *p* = 0.02), access‐site hematoma (1.2% vs. 3.3%; *p* = 0.01), and venipuncture (3.6% vs. 12.1%; *p* < 0.00001) in femoral cardiac procedures [11]. Another meta‐analysis published in 2024 of four randomized trials also demonstrated a significant benefit of ultrasound‐guided femoral access for coronary procedures in reducing major vascular complications or major bleeding (2.8% vs. 4.5%; *p* = 0.026) [9]. Subgroup analyses highlight benefits in high‐risk cohorts—obese patients, those on anticoagulation, in those requiring a large sheath, and in cases that received a closure device. It is crucial to acknowledge that the literature lacks a standardized method for ultrasound‐guided femoral access. Each study employs its own protocol or allows the technique to be determined at the discretion of the operator. What we propose in this document is a standardized step‐by‐step approach to facilitate the adoption of ultrasound‐guided CFA puncture by operators in their initial learning curve phase.

## 7 Conclusion

Ultrasound‐guided CFA puncture, executed via the proposed systematic six‐step protocol, can be an invaluable tool that facilitates achieving a “perfect” puncture. This approach has the potential to mitigate vascular complications, enhance compatibility with closure devices, and improve procedural efficiency. Though a learning curve exists, mastery transforms femoral access into a rapid, predictable, and safe process. Adoption in the catheterization laboratory, supported by equipment availability and training, is feasible and aligns with evidence demonstrating reduced complications in transfemoral cardiac and noncardiac procedures. As interventional cardiology evolves, standardizing ultrasound guidance for femoral access promises to elevate patient outcomes, reinforcing its role as a cornerstone of modern practice.

## Conflicts of Interest

The authors declare no conflicts of interest.

## Supporting information

Video 1 US anatomy assessment.

Video 2 CFA puncture and guide insertion.

Video 3 Single hand puncture and guidewire insertion.
